# Short-Term Outcomes of Three Consecutive Monthly Loading Administrations of Aflibercept 8 Mg for Treatment-Naïve Exudative Age-Related Macular Degeneration

**DOI:** 10.3390/ph18030438

**Published:** 2025-03-20

**Authors:** Shuhei Hosoda, Yoichi Sakurada, Yoshiko Fukuda, Yumi Kotoda, Wataru Kikushima, Kenji Kashiwagi

**Affiliations:** Department of Ophthalmology, Faculty of Medicine, University of Yamanashi, Yamanashi 409-3898, Japan; hosodas@yamanashi.ac.jp (S.H.); ysugiyama@yamanashi.ac.jp (Y.F.); ykanai@yamanashi.ac.jp (Y.K.); wkikushima@yamanashi.ac.jp (W.K.); kenjik@yamanashi.ac.jp (K.K.)

**Keywords:** exudative age-related macular degeneration, polypoidal choroidal vasculopathy, aflibercept 8 mg, short-term outcome

## Abstract

**Background/Objectives:** The aim was to investigate the short-term outcomes of three consecutive monthly aflibercept 8 mg administrations for treatment-naïve eyes with exudative age-related macular degeneration (AMD). **Methods:** Twenty-one eyes with exudative AMD were included (type 1 macular neovascularization: eleven eyes; type 2 macular neovascularization, four eyes; and polypoidal choroidal vasculopathy (PCV), six eyes). All eyes received three consecutive monthly administrations of aflibercept 8 mg (114.3 mg/mL) at an injection volume of 0.07 mL. Indocyanine green angiography (ICGA) was performed on eyes with PCV at baseline and at the 3-month visit. **Results:** The best-corrected visual acuity significantly (BCVA) improved from 0.31 ± 0.38 (baseline) to 0.25 ± 0.38 at the 3-month visits (*p* = 0.035). Dry macula achieved 62% and 100% at the 1-month and 3-month visits, respectively. Central retinal thickness and subfoveal choroidal thickness significantly decreased by 55.7% and 19.8%, from 341 ± 112 (baseline) to 190 ± 64 (3-month visits) and from 192 ± 50 (baseline) to 154 ± 51 (3-month visits), respectively (both *p* < 0.001). Complete regression of polypoidal lesions was seen in five (83.3%) eyes out of six on ICGA at the 3-month visit. No systemic adverse events were noted, and one eye developed a retinal pigment epithelial tear one month after the first injection. **Conclusions:** Three consecutive monthly administrations of aflibercept (8 mg) were safe and effective for resolving exudation and polyp regression, with significant BCVA improvement in treatment-naïve eyes with exudative AMD.

## 1. Introduction

Age-related macular degeneration (AMD) is the leading cause of blindness in developed countries. The number of patients is estimated to reach 200 million worldwide by 2040 [1]. The AMD stage is generally defined by the size of the drusen, that is, early, intermediate, and advanced stages [2,3]. However, the progression to advanced AMD varies depending on the drusen type [2,3,4,5]. Advanced AMD is classified into exudative AMD and geographic atrophy (GA) [4,5]. The incidence of GA, a rare phenotype of advanced AMD in the Asian population, is reported to be less than 10% in advanced AMD. Exudative AMD is characterized by macular neovascularization (MNV) developing subretinal hemorrhage and exudation including subretinal/intraretinal fluid, and it causes irreversible vision loss. The vascular endothelial growth factor (VEGF) is a pivotal pathogenesis for exudative AMD.

In 1999, the first large-scale randomized study, named TAP (Treatment of Age-related macular degeneration with photodynamic therapy), was reported in exudative AMD treated with photodynamic therapy (PDT) using 609 cohorts [6]. In the TAP study, eyes were assigned to the verteporfin–PDT group and the sham group. During the 24-month follow-up, the best-corrected visual acuity (BCVA) in the PDT group and sham group declined by 13.4 letters and 19.6 letters, respectively. PDT played a role in slowing the BCVA decline when compared with natural history. In the early 2000, pegaptanib was commercially available as the first VEGF inhibitor and was designed to block the VEGF-A 165 isoform of the five isoforms in the VEGF-A. In a randomized clinical trial, the VISION study, three different concentrations (0.3 mg, 1.0 mg, and 3.0 mg) of pegaptanib were intravitreally administrated every 6 weeks up to 54 weeks; however, BCVA gradually declined and could not maintain the baseline levels at 54 weeks irrespective of its concentration [7]. Ranibizumab, the second commercially available VEGF inhibitor with a molecular size of 48 k Da which was designed to block all VEGF-A isoforms, drastically changed the treatment of AMD [8]. In the MARINA and ANCHOR trials, monthly intravitreal administration of ranibizumab (0.5 mg/0.5 mL or 0.3 mg/0.5 mL) was effective in improving best-corrected visual acuity (BCVA) of 5.4–6.6 letters and 8.5–11.3 letters during the 24-month follow-up in eyes with exudative AMD irrespective of MNV subtypes. However, monthly dosing is an economic and physical burden for patients and physicians. In the PRONTO study, patients received additional injections based on the following three criteria after three consecutive monthly loading administrations of ranibizumab. These criteria were (1) exudation seen on optical coherence tomography (OCT), (2) increased central retinal thickness (CRT) by 100 μm on OCT, and (3) decline of five letters or more. In this trial, BCVA improved by 11.1 letters, CRT decreased by 212 μm, and the mean number of additional injections was 7.0 during a 24-month follow-up. This treatment regimen was called pro re nata or as-needed regimen. However, the SEVEN UP study, the extension study of intravitreal administration of ranibizumab treatment for naïve exudative AMD, ANCOH, MARINA, and the HORIZON study reported that the mean BCVA maintained during the 24-month follow-up gradually declined and returned to baseline levels 5 years after switching the as-needed regimen from 24-month monthly dosing, although one-third of the eyes demonstrated good visual outcomes, suggesting that the as-needed regimen is insufficient for certain patients. Subsequently, an 8-week interval administration of aflibercept (2 mg/0.05 mL or 0.5 mg/0.05 mL), the third commercially available VEGF inhibitor which is designed to bind all VEGF-A isoforms, VEGF-B, and placental growth factors with a molecular size of 97–115 k Da, enabled equivalent visual gains, namely 6.9–10.9 letters, to monthly ranibizumab administration during the 52-week follow-up, irrespective of MNV subtypes, in the VIEW 1 and 2 studies [9]. To date, intravitreal VEGF inhibitor administration monotherapy has been a standard treatment for exudative AMD, except for a specific AMD subtype, polypoidal choroidal vasculopathy (PCV) or pachychoroid neovasculopathy [10]. In eyes with pachychoroid spectrum disorders, including PCV and pachychoroid neovasculopathy, choroidal vascular hyperpermeability has been seen on indocyanine green angiography, and photodynamic therapy is effective for decreasing the choroidal thickness and resolution of exudation, with or without VEGF inhibitors. The prescription of VEGF inhibitors is increasing year-by-year in developed countries, including Japan, and medical expenses are a social problem and burden in these countries [11,12]. Therefore, the advent of long-acting drugs is to be expected.

Brolucizumab, the fourth commercially available VEGF inhibitor, was approved for use in the treatment of exudative AMD by the Food and Drug Administration (FDA) in 2020. Out of the commercially available VEGF inhibitors, brolucizumab has the smallest molecular size, with 26 k Da, and can achieve stability and solubility at a high dose in a single 50 μm intravitreal administration. At a dose of 6 mg, the equivalent molecular dose of brolucizumab is approximately 10 times greater than aflibercept and approximately 20 times greater than ranibizumab [13]. In the HAWK and HARRIER phase 3 clinical trials, patients were assigned to the treatment of brolucizumab (6.0 mg/0.05 mL), brolucizumab (3.0 mg/0.05 mL), and aflibercept (2.0 mg/0.05 mL). In eyes treated with brolucizumab (6.0 mg/0.05 mL), almost 60% of eyes could continue the 12-week interval administration after three monthly loading administrations during the 44 weeks of follow-up in the unconventional treatment regimen, obtaining a similar visual gain of 6.6 letters compared to 8-week interval aflibercept (2.0 mg/0.05 mL) administration after three consecutive monthly administrations. At 96 weeks, 45.4% and 38.6% of eyes treated with brolucizumab (6.0 mg/0.05 mL) could continue the 12-week interval in the HAWK and HARRIER trials, respectively [14]. In 2022, faricimab, the fifth commercially available inhibitor, was approved for the treatment of exudative AMD by the FDA. Faricimab, a fully human 150 k Da monoclonal antibody of bispecific design, concurrently targes VEGF-A and angiopoietin 2 (Ang-2), disrupting two pivotal signaling pathways in the pathogenesis of AMD. This unique bispecific antibody binds both VEGF-A and Ang-2 and offers a better therapeutic effect compared to VEGF-A inhibition only. TENAYA and LUCERN, phase 3 randomized double-masked trials performed worldwide, compared 8-week interval aflibercept administration with personalized intervals of faricimab treatment in exudative AMD [15]. This personalized treatment regimen could extend the interval by up to 16 weeks. In both the TENAYA and LUCERN trials, faricimab (6.0 mg/0.05 mL) demonstrated non-inferior BCVA improvement of 5.5–6.5 letters compared to the aflibercept 2.0 mg treatment group at 48 weeks. In both trials, approximately 80% of patients receiving faricimab treatment could extend administration over a 12-week treatment interval. A total of 45% of the patients attained the longest dosing interval, namely every 16 weeks, by the end of the first year, suggesting the long-lasting effect of faricimab treatment. In both the TENAYA and LUCERN trials, patients treated with faricimab were followed by the treat-and-extended regimen from 60 weeks to 108 weeks. In the treatment phase during the 60–108 weeks, the median number of injections in group receiving the faricimab treatment was three, and a BCVA improvement of 3.7–5.0 letters was maintained at 108 weeks [16]. Thus, VEGF inhibitors are expected to have longer-duration exudation suppression to reduce the burden on patients and physicians and medical expenses.

Brolucizumab and faricimab could prolong the treatment interval owing to their high molecular concentrations in a single intravitreal injection and offered similar visual improvement compared to aflibercept 2 mg in clinical trials and real-world studies [15,17]. Therefore, they were categorized as second-generation VEGF inhibitors.

Recently, a pivotal randomized clinical trial, the PULSAR study, investigated the efficacy and safety of 8 mg aflibercept for exudative AMD compared to aflibercept 2 mg. In this study, eyes were assigned to three treatment groups—(1) administration of aflibercept 2 mg every 8 weeks, (2) aflibercept 8 mg every 12 weeks, and (3) aflibercept 8 mg every 16 weeks—following consecutive 3-monthly administration. During the follow-up periods, if eyes failed to meet the prespecified dose regimen modification criteria, the aflibercept 8 mg treatment group had their dosing interval shortened. In this study, aflibercept 8 mg was maintained in 79–88% of patients at least over a 12-week administration interval, with non-inferior visual improvement of 6.2–6.7 letters in the ETDRS chart compared to aflibercept 2 mg at the 48-week visit [18].

Second-generation VEGF inhibitors are considered to be comparable to first-generation VEGF inhibitors, including ranibizumab and aflibercept 2 mg, in terms of the incidence of systemic adverse events. However, topical adverse events, mainly intraocular inflammation (IOI), are a concern when intravitreal second-generation VEGF inhibitors are administrated. In HAWK and HARRIER, the incidence of IOI was higher in brolucizumab 6 mg at 4% than in aflibercept 2.0 mg at 1%. In other second-generation VEGF inhibitors, IOI was reported in the real world [19,20,21]. Depending on the inflammation origin, IOI was classified into anterior/middle or posterior segments. In general, the prognosis of anterior/middle segment IOI is favorable when topical corticosteroids are administrated. On the other hand, the prognosis of the posterior segment is generally poor, especially when presenting with retinal vascular occlusion with or without retinal vasculitis, even if adequate treatment comprising topical and systemic corticosteroids is administrated. Several causes are speculated for developing IOI, but the precise mechanism has not been understood. A high VEGF concentration is considered one of the causes of IOI development [22].

Aflibercept 8 mg is the third commercially available second-generation VEGF inhibitor, circulated in 2024, following brolucizumab and faricimab; however, limited information regarding the loading phase of aflibercept 8 mg for exudative AMD is available. In the present study, we report the short-term outcomes of three consecutive monthly doses of aflibercept 8 mg for exudative AMD in the Japanese population.

## 2. Results

**Figure 1:**
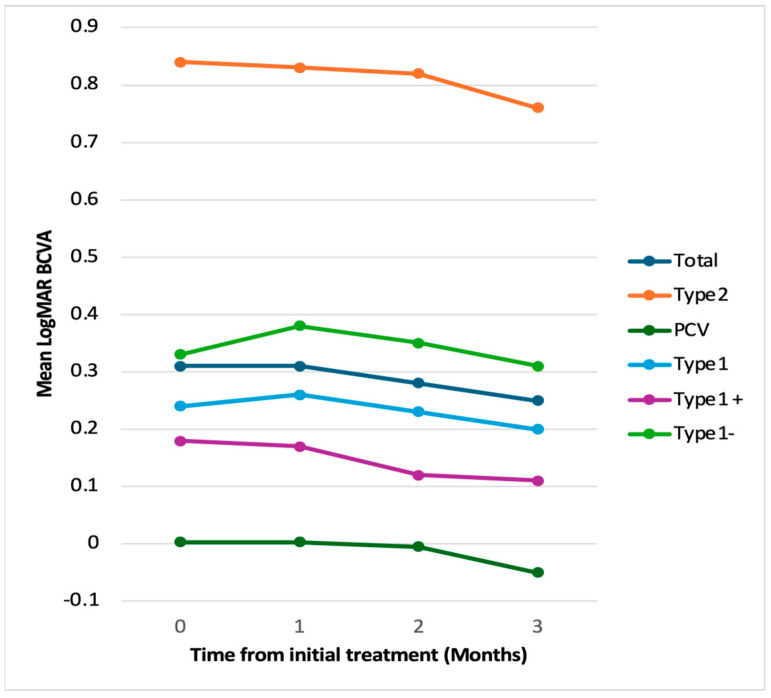
Change in BCVA after treatment according to exudative AMD subtype. In 21 eyes, the best-corrected visual acuity (BCVA) improved from 0.31 ± 0.38 to 0.25 ± 0.38 at the 3-month visit. In eyes with type 1 MNV, the BCVA improved from 0.24 ± 0.21 to 0.20 ± 0.22 at the 3-month visit. In eyes with type 1 MNV and fibrovascular PED, the BCVA improved from 0.18 ± 0.15 to 0.11 ± 0.11 at the 3-month visit. In eyes with type 1 MNV without fibrovascular PED, the BCVA improved from 0.33 ± 0.23 to 0.31 ± 0.25 at the 3-month visit. In eyes with PCV, the BCVA improved from 0.00 ± 0.47 to −0.05 ± 0.49 at the 3-month visit.

**Figure 2:**
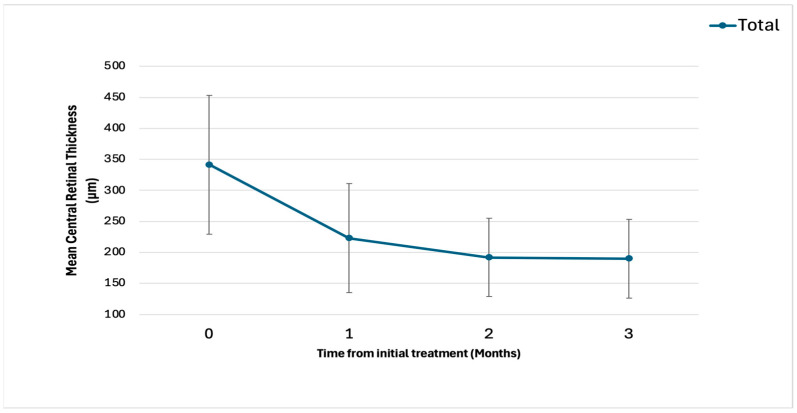
Change in central retinal thickness (CRT) after treatment. Baseline CRT significantly decreased from 341 ± 112 to 223 ± 88, 192 ± 63, and 190 ± 64 μm at the 1-month, 2-month, and 3-month visits, respectively (all, *p* < 0.001).

**Figure 3:**
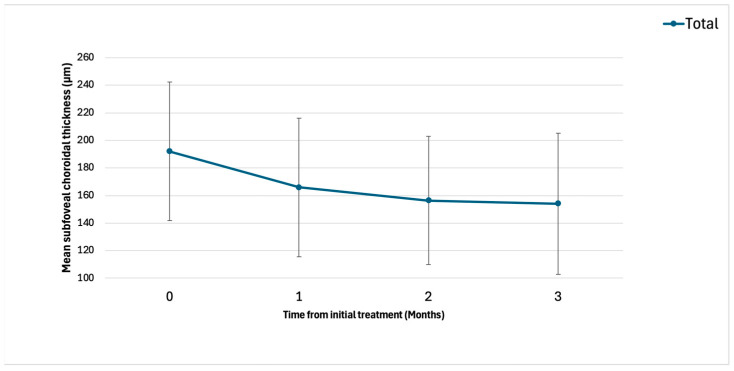
Change in subfoveal choroidal thickness (SCT) after treatment. Baseline SCT significantly decreased from 192 ± 50 to 166 ± 50, 156 ± 47, and 154 ± 51 μm at the 1-month, 2-month, and 3-month visits, respectively (all, *p* < 0.001).

**Figure 4:**
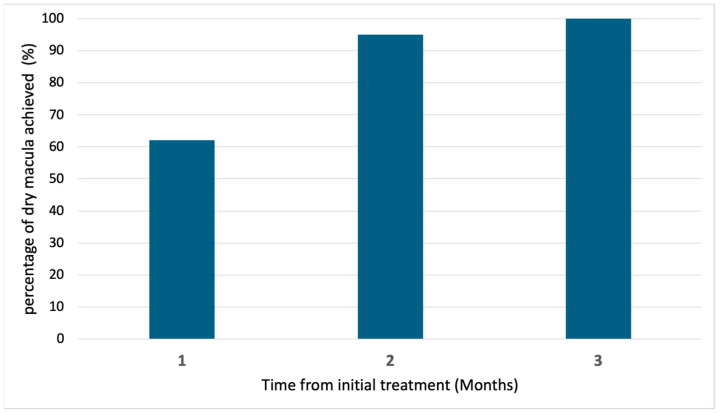
Dry macula rate evaluated by optical coherence tomography after treatment. Dry macula was observed in 62%, 95%, and 100% of cases at the 1-month, 2-month, and 3-month visits, respectively.

**Figure 5:**
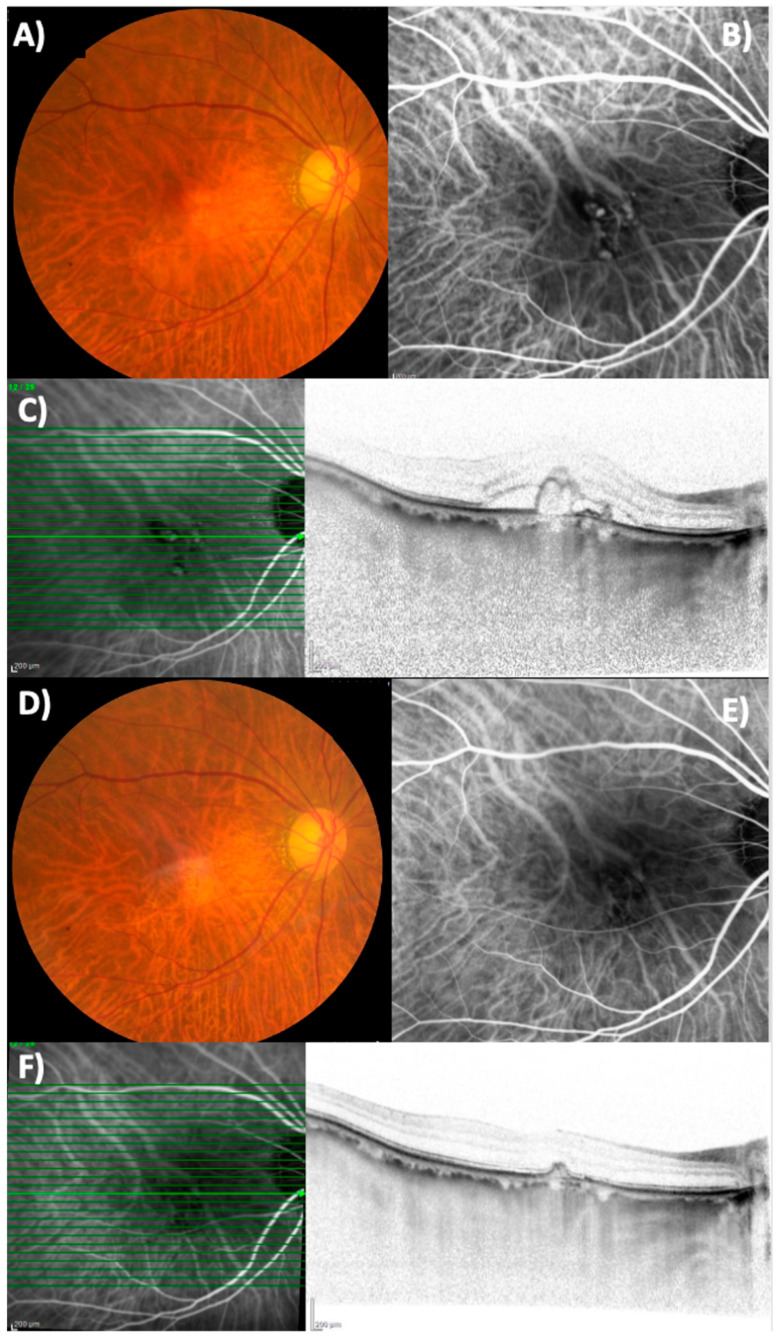
A 74-year-old male with polypoidal choroidal vasculopathy in the right eye. (**A**) A retinal pigmentary epithelial change can be seen in the macula in the right eye. (**B**) Indocyanine green angiography (ICGA) shows a branching vascular network with polypoidal lesions. (**C**) Spectral-domain optical coherence tomography (SD-OCT) reveals subretinal fluid and protrusion of the retinal pigment epithelium (RPE), corresponding to a horizontal line through a polyp. (**D**) In the right eye, pigmentary changes persisted for a month after three consecutive monthly administrations of aflibercept 8 mg. (**E**) Complete regression of the polyps was observed on ICGA one month after the third injection. (**F**) The RPE bump remained on the horizontal SD-OCT scan despite complete polyp regression on ICGA.

The baseline demographic and clinical characteristics are shown in Table 1. Exudative AMD can be classified into four subtypes: type 1, 2, and 3 macular neovascularization (MNV) and PCV. In this study, none of the eyes contained type 3 MNV. Figure 1 shows the changes in the BCVA after treatment. Type 1 MNV was further subdivided into those with and without fibrovascular pigment epithelial detachment (PED). In 21 eyes, best-corrected visual acuity (BCVA) improved from 0.31 ± 0.38 (baseline) to 0.25 ± 0.38 (3-month visit). In 11 eyes with type 1 MNV, BCVA improved from 0.24 ± 0.21 (baseline) to 0.20 ± 0.22 (3-month visit). In four eyes with type 2 MNV, BCVA improved from 0.84 ± 0.48 (baseline) to 0.76 ± 0.49 (3-month visit). In six eyes with PCV, BCVA improved from 0.00 ± 0.47 (baseline) to −0.05 ± 0.49 (3-month visit). Figure 2 and Figure 3 show the changes in the central retinal thickness (CRT) and subfoveal choroidal thickness (SCT). CRT and SCT significantly decreased at the 1-, 2-, and 3-month visits (*p* < 0.001). The dry macular rate after treatment is shown in Figure 4. In eyes with PCV, complete regression of polypoidal lesions was observed in five (83%) out of six eyes. Representative images of the complete regression are shown in Figure 5.

Regarding local adverse events, a retinal pigment epithelial (RPE) tear was observed in one female patient with fibrovascular PED one month after the first injection. None of the patients developed intraocular inflammation during this study.

## 3. Discussion

In the present study, we investigated the short-term outcomes of three consecutive 3-monthly aflibercept 8 mg administrations for treatment-naïve eyes with exudative AMD. BCVA significantly improved at the 3-month visit, and a dry macula was achieved in all patients, with a significant reduction in central retinal thickness (CRT) and subfoveal choroidal thickness (SCT) irrespective of the MNV subtype. In the eyes with PCV, complete regression was seen in 83% of the eyes with PCV on ICGA one month after three consecutive monthly administrations of aflibercept 8 mg.

The VIEW study reported a 70% dry macula at 8 weeks after three consecutive 4-weekly intravitreal injections of aflibercept 2 mg [9]. Although the OCT scan protocols differed from those of the previous study, dry macula was found in 62% and 100% of patients 1 month after the first and third aflibercept 8 mg administrations, respectively, in the present study. This suggests that the three consecutive monthly administrations of aflibercept 8 mg were effective for the resolution of exudation, including subretinal and intraretinal fluid, in treatment-naïve eyes with exudative AMD.

Subfoveal choroidal thickness decreased by 19.8% one month after the third aflibercept 8 mg administration. In other second-generation VEGF inhibitors, including brolucizumab and faricimab, subfoveal choroidal thickness decreased by 15–20% and 10–12% one month after three consecutive monthly administrations of brolucizumab and faricimab, respectively [23,24,25,26]. In this study, the extent of SCT reduction was similar to that in previous reports demonstrating a reduction in SCT one month after the three-monthly administration of brolucizumab. Like SCT, CRT significantly decreased at all time points, including at 1, 2, and 3 months.

A few studies have demonstrated that the complete regression of polyps after loading administration leads to a better long-standing treatment response [27,28]. Sayanagi et al. reported that eyes with complete regression of polyps had significantly better visual improvement, fewer additional treatments, and fewer recurrences compared with eyes in which polyp regression did not occur. In this study, complete regression of polyps was seen in five (83%) out of six eyes one month after the third administration of aflibercept 8 mg upon indocyanine green angiography. Previous studies had demonstrated that the complete polyp regression rate was 30–55%, 52–61%, and 56–87% one month after the loading administration of aflibercept 2 mg, faricimab, and brolucizumab, respectively, in eyes with PCV [23,24,25,26]. The number of eyes with PCV was small in this study, but the effect of complete regression might have been stronger than that obtained with aflibercept. There are possible reasons for the higher rate of resolving exudation and complete regression of polyps. VEGF is a pivotal stimulator of angiogenesis and vascular hyperpermeability which promotes the development and progression of macular/retinal neovascularization and exudation including subretinal and intraretinal fluid. As a VEGF inhibitor, a dose of aflibercept 8 mg four times compared to aflibercept 2 mg theoretically has a stronger effect on the resolution of exudation and the regression of macular neovascularization including polyps [29].

In this study, BCVA significantly improved from 0.31 ± 0.28 to 0.25 ± 0.38 in 21 eyes with exudative AMD. However, the extent of BCVA improvement was smaller than that observed in the PULSAR study. One explanation for this finding is better baseline visual acuity. The BCVA inclusion criteria in the PULSAR trial were between 20/320 and 20/32, and the mean BCVA was approximately 60 letters, corresponding to 0.32 in a decimal format. Our cohort’s baseline mean BCVA was log MAR 0.31, corresponding to 0.5 in decimal format. Owing to the ceiling effect, the improvement in BCVA might have thus been limited.

This study had several limitations. First, the sample size was small. Although BCVA significantly improved in 21 eyes, we could not conduct an analysis according to the AMD subtype. Secondly, regarding the high rate of complete regression of polypoidal lesions, a large cohort study is necessary to validate these outcomes. Recently, we investigated the 12-month remission rate after consecutive 3-monthly aflibercept 2.0 mg for exudative AMD using an as-needed regimen during the 60-month follow-up. In this study, 57% of patients showed 12-month remission or more at least once during the 60-month follow-up [30]. It might be interesting to compare the rate of 12-month remission between aflibercept 2.0 mg and 8.0 mg over a long-term follow-up.

## 4. Methods

A retrospective study was conducted on consecutive patients with treatment-naive exudative AMD who initiated three consecutive monthly 0.07 mL administrations of 8 mg aflibercept (114.3 mg/mL) in the vitreous humor between 1 July 2024 and 31 October 2024. This retrospective study was approved by the Institutional Review Board of the University of Yamanashi and performed according to the tenets of the Declaration of Helsinki (approval no. 2892). Before treatment initiation, each patient provided their written informed consent.

During the initial visit, all patients received a comprehensive ophthalmic examination, including best-corrected visual acuity measurements using a Landolt chart, slit-lamp biomicroscopy with or without a 78-diopter lens, intraocular pressure measurement, fundus photography, swept-source (SS) or spectral-domain (SD) optical coherence tomography (SS-OCT/SD-OCT) using Atlantis (Topcon, Tokyo, Japan) and Spectralis (Heidelberg Engineering, Dossenheim, Germany), and fluorescein and indocyanine green angiography (FA/ICGA) using a confocal laser scanning system (HRA-2).

All patients were followed up monthly from the baseline to the 3-month visits. During the follow-up visits at 1, 2, and 3 months, all patients underwent examinations, including measuring BCVA using a Landolt chart, biomicroscopy with or without a 78-diopter lens, measuring intraocular pressure, color fundus photography, and OCT as performed at the initial visit. At three months, FA/ICGA was also performed on eyes with polypoidal choroidal vasculopathy (PCV) to confirm the presence or absence of polypoidal lesions.

Intravitreal aflibercept (114.3 mg/mL) was administered sterilely at a 0.07 mL dose to the vitreous cavity at baseline, 1 month, and 2 months in a sterile fashion.

SS-OCT measured the central retinal thickness as the vertical distance between Bruch’s membrane and the inner limiting membrane at the fovea. The subfoveal choroidal thickness was defined as the vertical distance between the chorioscrelal border and Bruch’s membrane at the fovea, as measured using SS-OCT.

A dry macula was defined as the complete resolution of the subretinal and intraretinal fluid, as measured using SS-OCT. The SS-OCT protocol consisted of a crosshair scan of the fovea with a length of 9 mm.

Statistical analyses were conducted using the DR. SPSS (V.30). A paired *t*-test was performed to evaluate the differences between the baseline values and those at 1, 2, and 3 months. Statistical significance was set at *p* < 0.05.

## 5. Conclusions

In conclusion, three consecutive monthly aflibercept 8 mg administrations are safe and effective for the anatomical response, including achieving a dry macula and the complete regression of polyps.

## Figures and Tables

**Table 1:** Baseline characteristics in patients with exudative age-related macular degeneration.

Characteristics (N = 21)	Values
Age (years)	75 ± 9.9
Sex Male/Female (%)	11 (52)/10 (48)
R/L (%)	7 (33)/14 (67)
Phakia/IOL (%)	16 (76)/5 (24)
BCVA (logMAR)	0.31 ± 0.38
CRT (μm)	341 ± 112
SCT (μm)	192 ± 50
Type1 MNV/Type2 MNV/PCV (%)	11 (52)/4 (19)/6 (29)

BCVA: best-corrected visual acuity; CRT: central retinal thickness; IOL: intraocular lens; logMAR: logarithm of the minimum angle of resolution; MNV: macular neovascularization; and SCT: subfoveal choroidal thickness.

## Data Availability

Data are contained within the article.
